# Laparoscopic resection of hepatic alveolar echinococcosis: A single-center experience

**DOI:** 10.1371/journal.pntd.0010708

**Published:** 2022-09-06

**Authors:** Severin Gloor, Daniel Candinas, Guido Beldi, Anja Lachenmayer

**Affiliations:** Department of Visceral Surgery and Medicine, Inselspital, Bern University Hospital, University of Bern, Switzerland; University of Ulm, GERMANY

## Abstract

**Introduction:**

Alveolar echinococcosis (AE) remains a very rare disease requiring complete radical resection for curative treatment. While open approaches are common, safety and efficacy of laparoscopic resections remain unknown.

**Methods:**

This is a single-center, retrospective cohort study with patients undergoing liver resection for hepatic AE at the Department of Visceral Surgery and Medicine, Bern University Hospital from December 2002 to December 2020. Postoperative outcomes of patients following laparoscopic hepatectomy (LH) for hepatic AE were compared with those of patients undergoing open hepatectomy (OH).

**Results:**

A total of 93 patients underwent liver resection for hepatic AE. Laparoscopic hepatectomy was performed in 23 patients and open hepatectomy in 70 patients. While there were no significant differences in terms of gender, age and diagnostic tools, the majority of patients of the LH cohort were PNM stage 1 (78%) in contrast to only 39% in the OH cohort (*p = 0*.*002*). Patients undergoing laparoscopic hepatectomy were treated by minor liver resections in 91% and in 9% by major liver resections in comparison to the open hepatectomy cohort with 61% major liver resections and 39% minor resections. Laparoscopic hepatectomy was associated with shorter mean operation time (127 minutes vs. 242 minutes, *p <0*.*001*), lower major complication rate (0% vs. 11%, *p = 0*.*322*) and shorter mean length of hospital stay (4 days vs. 13 days, *p <0*.*001*). Patients with LH had a distinct, but not significant lower recurrence rate (0% vs. 4%, *p = 0*.*210*) during a mean follow-up of 55 months compared with a follow-up of 76 months in the OH cohort. After subgroup analysis of PNM stage 1 patients, similar results are seen with persistent shorter mean operation time (120 minutes vs. 223 minutes, *p <0*.*001*), lower major complication rate (0% vs. 8%, *p = 0*.*759*) and shorter length of hospital stay (4 days vs. 12 days, *p <0*.*001*).

**Conclusion:**

Laparoscopy appears as a feasible and safe approach for patients with PNM stage 1 alveolar echinococcosis without impact on early disease recurrence.

## Author summary

In this retrospective cohort study laparoscopic hepatectomy for hepatic alveolar echinococcosis had no negative impact on perioperative outcomes, disease recurrence or survival compared with open hepatectomy. The importance of this finding is that the laparoscopic approach is feasible and safe for selected patients with hepatic alveolar echinococcosis, especially those with PNM stage 1.

## Introduction

Echinococcus multilocularis is the causative agent of alveolar echinococcosis (AE) which predominantly affects the liver [1]. Only in 2.3% of cases other organs (e.g. diaphragm, peri-renal tissue, lymph nodes, peritoneum, lung, brain, spleen or bone) become initially infected by the larval stages (metacestodes) of the parasite [2,3]. Although its overall incidence remains low, certain endemic regions report increasing numbers including Switzerland with an incidence of 0.3 to 3/million inhabitants/year [2,4].

About 70% of patients typically present with symptoms such as hepatobiliary complications or abdominal pain, while one third of patients is usually detected incidentally [5,6]. With the increasing use of cross sectional imaging (abdominal sonography, computed tomography or magnetic resonance imaging), immunosuppressive medication and eventually also an altered infectious potential, diagnoses seem to increase in the recent years [4]. Further, serology (purified Echinococcus multilocularis Em2-ELISA, Echinococcus multilocularis recEm18-ELISA and Echinococcus granulosus EgHF-ELISA) of AE supports differentiation of hepatic lesions, enables to differentiate between alveolar and cystic echinococcosis [7] and to interpret the activity of the infection [8].

The only curative treatment available is a complete resection of the part of the liver containing the parasitic mass. Radical resection with open surgery is up to now the standard approach for hepatic AE-lesions ^5^ and several studies have shown safety and efficacy with rates of moderate and major complications (Clavien-Dindo ≥3a) between 2.7% and 15% [9–11]. While cystic echinococcosis is now increasingly operated laparoscopically [12], very few studies have reported feasibility and safety of laparoscopic and robotic resection in AE patients so far [13–15]. Although laparoscopic hepatic resections are now commonly used for hepatic malignancies with no differences in oncologic results tested on colorectal liver metastasis [16], not much is known about the safety and efficacy of laparoscopic surgery for AE. Especially still recommended safety margin of 2 cm in order to avoid disease recurrence because of its slow and progressive infiltrative growth into liver tissue and along the central bile ducts [17] inhibited progress of laparoscopic AE resection.

Current recommendations suggest to treat with Albendazole (ABZ) 2 years after complete surgical resection [5]. In patients with non-resectable disease lifelong ABZ-therapy offers excellent long-term survival [18,19] with a low incidence of severe adverse effects of ABZ-therapy such as alopecia, elevated liver enzymes, gastrointestinal disorders or leucopenia [1,20,21].

We therefore aimed to analyze safety and feasibility of laparoscopic resection for alveolar echinococcosis in our retrospective single-center analysis.

## Methods

### Ethics statement

This study was initiated after obtaining approval from the Ethics Commission of the Canton of Bern (KEK Nr. 2017–01534) and with an amendment for the time frame 2018–2020. General written informed consent was obtained from all subjects involved in the study.

### Patient inclusion criteria

This trial is a single-center, retrospective analysis of patients undergoing liver resections due to alveolar echinococcosis (AE) at the Department of Visceral Surgery and Medicine at the Inselspital, Bern University Hospital.

In our clinic a total of 155 patients were treated due to AE during the period of 2002–2020. The inclusion criteria contained adult patients over age 18, which underwent elective liver resection for AE. Patients not requiring biliary reconstructions or large anatomic resections such as hemihepatectomies, extended hemihepatectomies or central liver resections were operated laparoscopically if technically feasible. The cohort of 62 patients who were only treated with ABZ were excluded. None of the patients had documented objection to subsequent use of personal health data. The data of the conservatively treated patients has recently been published [22]. Clinicopathological data of 93 consecutive patients were extracted including demographics, radiological characteristics, therapeutic features, intraoperative findings, surgical procedures, pathological examination, morbidity and complications.

### Preoperative assessment

All patients suffering from AE were evaluated by an interdisciplinary team of hepatologists and hepatobiliary surgeons according to the standardized procedure (medical history, physical examination, laboratory tests, radiological exams and anesthesia evaluation) in our department. PNM stage was defined from radiological exams [23], either by computed tomography or magnetic resonance imaging. Serological testing of Echinococcus-specific antibodies (EgHF, Em2 and recEm18) was used as an additional examination to diagnose AE. Individual treatment for each patient was discussed in the weekly multidisciplinary liver boards attended by hepatobiliary surgeons, hepatologists, radiologists and pathologists. Hepatectomy was recommended if AE lesions could be resected with preservation of a sufficient future liver remnant (FLR). To assess lesion location in the liver and to estimate FLR contrast-enhanced computed tomography (CT) or magnetic resonance imaging (MRI) with liver-specific contrast agents was performed. If FLR was estimated smaller than 30% of liver volume, 3D-volumetry was added to calculate exact volumes and to define risk of liver insufficiency. Preoperative medical therapy with albendazole (ABZ) was administered according to the assessment of the multidisciplinary liver boards. Patients were excluded, if no curative surgical resection could be performed or patients refused the operation.

### Surgical procedure and postoperative management

Patients were treated by laparoscopy according to the preoperative decision of the surgeon which was based on the experience with laparoscopic liver surgery of primary or secondary liver malignancies [16]. The difficulty score for laparoscopic liver resection was calculated [24]. This score includes tumor location, extent of liver resection, tumor size, proximity to major vessel and the liver function and reflects difficulty of laparoscopic approach well. All liver resections were performed by AE-experienced and hepatobiliary surgeons also trained and experienced in oncologic laparoscopic liver surgery. Blood loss was calculated after suction of all intraabdominal liquid during operation, whereas flushing liquid was subtracted afterwards. Postoperative complications within 90 days after surgery were graded according to the classification of Clavien and Dindo [25]. Major morbidity was defined as any complication ≥3b grade and postoperative mortality as grade 5.

Patients received disease surveillance according to institutional standards and international guidelines at least annually [5]. Postoperative medical therapy with ABZ was administered according to the postoperative assessment of the multidisciplinary liver boards. AE recurrence was defined as the first lesion relapse following curative intended hepatectomy for AE combined with an increase of sampled antibodies in serology. Radiologists reviewed during the scheduled long-term disease surveillance cross-sectional imaging (CT-scan, MRI) to identify the presence and location of recurrent disease.

### Histological evaluation

Histology was performed upon resected specimens, yielding diagnosis and resection margin status of AE, as well as resection margin. R1 resection was defined as the microscopic presence and R2 as the macroscopic presence of the laminar layer within the transection line, whereas R0 resection was defined as complete resection with no laminar layer within 1 mm of the resection margin. In case of R2 resection any detected disease of AE during follow-up was classified as persistent.

### Serological testing

From all patients serum samples were collected to test perioperative Echinococcus-specific antibodies (EgHF, Em2 and recEm18). All three ELISAs were carried out as previously described [26]. Antibody levels against EgHF, Em2 and recEm18 were compared between last preoperative and first postoperative samples, which could vary due to missing international standards.

### Statistical analysis

Quantitative and qualitative variables were expressed as mean (standard deviation) and frequency (percentage). Number and stage of complications as well as overall survival (OS), recurrence-free survival (RFS), recurrence rate, length of medical therapy and mortality were analyzed. Comparisons between cohorts were analyzed with the chi-square or Fisher exact test for categorical variables and the Mann-Whitney U test for continuous variables, as appropriate. Using the Kaplan-Meier method, OS and RFS was calculated from the date of resection to the date of death, date of recurrence or last follow-up respectively. Comparisons between survival or recurrence rates were performed using log-rank tests. Survival time was calculated from the date of operation until the date of interest (last visit or death). P < 0.05 was considered statistically significant. Subgroup analyses were performed for patients with PNM stage 1. The subgroup is labelled with the prefix ‘PNM stage 1’. Statistical analysis was performed using SPSS version 25 (IBM, Armonk, New York, USA).

## Results

### Clinical data

Table 1 summarizes the clinical data of the 23/93 (25%) patients with laparoscopic hepatectomy (LH) and 70/93 (75%) with open hepatectomy (OH). While there were no significant differences in terms of gender, age and diagnostic tools, the majority patients of the LH cohort were PNM stage 1 (n = 18/23, 78%) in contrast to only 39% (n = 25/70) in the OH cohort (*p = 0*.*002*). The hepatic lesions in the LH cohort were located in one left-sided segment (n = 4/23, 17%), one central segment (n = 1/23, 4%), one right sided segment (n = 2/23, 9%), two left-sided segments (n = 2/23, 9%), two central segments (n = 2/23, 9%), two right-sided segments (n = 2/23, 9%), more than three right-sided segments (n = 3/23, 13%) and more than three bilateral segments (n = 7/23, 30%). In contrast, hepatic lesions of the OH cohort were located in one left-sided segment (n = 1/70, 1%), one central segment (n = 3/70, 4%), one right sided segment (n = 6/70, 9%), two left-sided segments (n = 5/70, 7%), two central segments (n = 2/70, 3%), two right-sided segments (n = 12/70, 17%), two bilateral segments (n = 2/70, 3%), more than three left-sided segments (n = 7/70, 10%), more than three central segments (n = 4/70, 6%), more than three right-sided segments (n = 13/70, 19%) and more than three bilateral segments (n = 15/70, 21%). The mean maximal diameter was significantly higher (8.6 cm vs. 4.3 cm, *p < 0*.*001*) in the OH cohort. Local extrahepatic lesions were more common in OH cohort (OH 20% [n = 14/70] vs. LH 4% [n = 1/23], *p = 0*.*042*) and consisted of infiltration to the diaphragm and the greater omentum. A distant extrahepatic disease to brain (n = 1) and peritoneum (n = 1) existed in two patients of the OH cohort (n = 2/70, 3%), compared to absent extrahepatic disease in the LH cohort (*p = 0*.*408*). The interdisciplinary tumor board has recommended a resection of that brain metastasis due to neurological symptoms after primary hepatectomy. The other patient received a tumor debulking surgery in palliative intention because he suffered from severe edemas of the lower limbs and massive ascites due to an external compression of the inferior vena cava and the portal vein caused by massive peritoneal AE, respectively. Further there was an insignificant difference for the mean time from diagnosis to resection, which was shorter in the OH cohort (4 months vs. 5 months, *p = 0*.*554*) (Table 1).

**Table 1:** Comparison of demographic and preoperative clinical data between laparoscopic and open resection of patients with hepatic alveolar echinococcosis.

Variable	LH (n = 23)	OH (n = 70)	*p-value*
Gender, n (%)			*0*.*852*
Female	13/23 (57)	38/70 (54)	
Male	10/23 (43)	32/70 (56)	
Age, mean years (SD)	57 (±17)	53 (±15)	*0*.*193*
Primary radiologic examination, n (%)			
Sonography	3/23 (13)	9/70 (13)	*0*.*982*
CT-scan	14/23 (61)	43/70 (61)	*0*.*962*
MRI	4/23 (17)	8/70 (11)	*0*.*462*
Unknown	2/23 (9)	10/70 (15)	*0*.*490*
PNM-stage, n (%)			
1	18/23 (78)	25/70 (39)	*<0*.*001*
2	0/23 (0)	11/70 (16)	*0*.*044*
3a	3/23 (13)	10/70 (14)	*0*.*882*
3b	2/23 (9)	14/70 (20)	*0*.*215*
4	0/23 (0)	7/70 (10)	*0*.*117*
Unknown	0/23 (0)	3/70 (4)	*0*.*315*
Maximal diameter of lesion, mean cm (SD)	4.3 (±3.1)	8.6 (±4.7)	*<0*.*001*
Local extrahepatic lesions, n (%)	1/23 (4)	14/70 (20)	*0*.*042*
Distant extrahepatic lesions, n (%)	0/23 (0)	2/70 (3)	*0*.*408*
Preoperative treatment with albendazole, n (%)	16/23 (70)	51/70 (73)	*0*.*392*
Time from diagnosis to operation, mean months (SD)	4 (±5)	5 (±13)	*0*.*554*

CT, computed tomography; LH, Laparoscopic hepatectomy; MRI, magnetic resonance imaging; OH, open hepatectomy; PNM, PNM system (P = parasitic mass in the liver, N = involvement of neighbouring organs, and M = metastasis); SD, standard deviation

### Perioperative data

**Fig 1:**
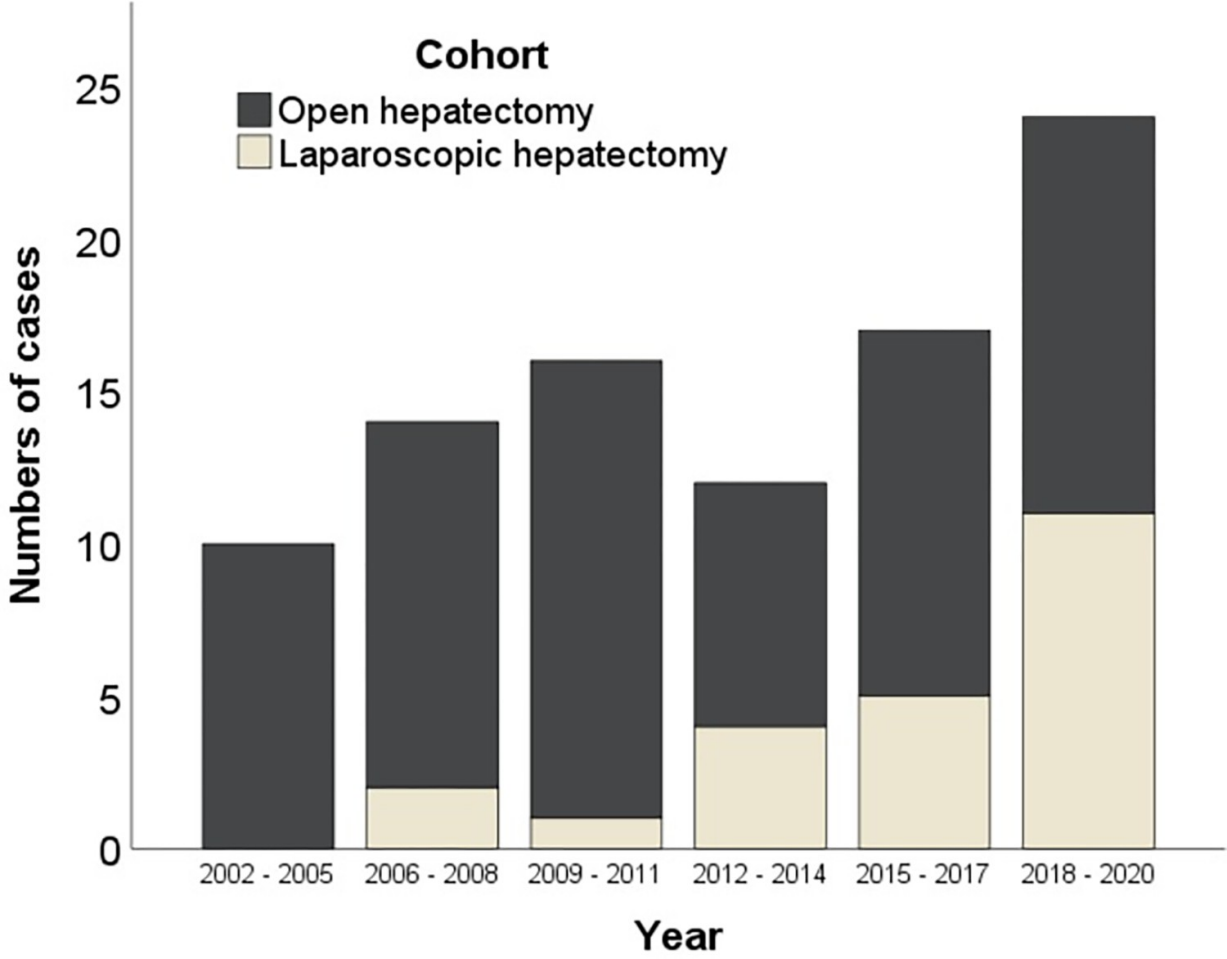
Development of caseload in AE cohort.

**Fig 2:**
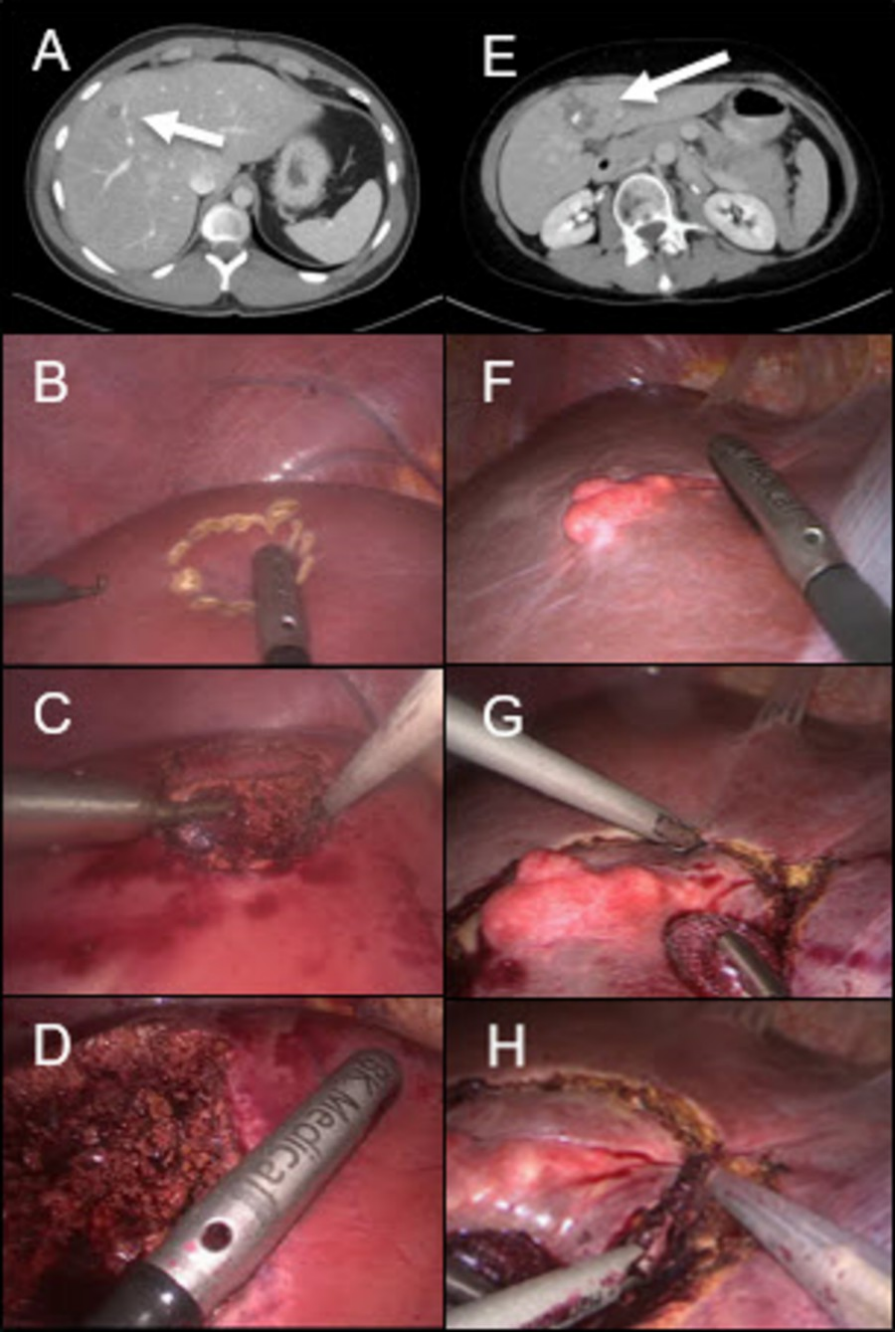
Laparoscopic resection of AE-lesions in two patients: In the first patient, CT-scan shows a small lesion in central liver segment 4a/b (A). Prior to resection an intraoperative sonography was made and the lesion was marked (B). The dissection of liver parenchyma was performed by an ultrasonic dissection device (C). A post-resectional sonography showed free resection margins (D). The CT-scan of a second patient shows a calcified lesion in liver segment 4b (E). This lesion could be macroscopically seen in the diagnostic laparoscopy (F). Dissection of liver parenchyma was done by an ultrasonic dissection device (G) and by ultrasonic surgical aspirator system (H).

The majority of patients in the LH cohort were treated by minor hepatic resections (n = 21/23, 91%) including bi-segmentectomies (n = 4/23, 17%), segmentectomies (n = 1/23, 4%) or atypical liver resections (n = 16/23, 70%) in comparison to the OH cohort with significant more major liver resections and only 39% (n = 27/70) minor resections (13% bi-segmentectomies [n = 9/70], 6% segmentectomies [n = 4/70] or 20% atypical liver resections [n = 14/70]). After the first laparoscopic AE resection at our center in 2006 the caseload increased steadily within the years (Fig 1). According to the difficulty score for laparoscopic liver resection a mean score of 5.05 (SD ±2.8) points in the LH cohort was seen [24]. The mean operation time was significantly shorter in the LH patients compared with OH patients (127 minutes vs. 242 minutes, *p <0*.*001*) and LH patients have lost less blood during operation (307 ml vs. 694 ml, *p = 0*.*003*). Conversion from laparoscopic to open approach was needed in two patients due to technical issues in case of tumor extent across the central bile duct in one case and the first portal pedicle in the other case requiring open dissection and biliary reconstruction. Fig 2 shows two examples of laparoscopic AE resections.

### Complications and hospital stay

The mean length of stay in the LH cohort was significantly shorter (4 days) than in the OH cohort (13 days, *p < 0*.*001*) (Table 2). The LH cohort had a significantly lower overall complication rate (9% [n = 2/23] vs. 43% [n = 30/70], p *= 0*.*003*) and lower major complication rate (0% [n = 0/23] vs. 11% [n = 8/70], *p = 0*.*322*). In the LH cohort the only minor complication of grade 3a according to Clavien-Dindo requiring intervention was a biliary leakage from the resection area treated with percutaneous drain insertion and stenting of the biliary duct by endoscopic retrograde cholangiopancreaticography. In the OH cohort, 8 major complications occurred including five infected biliomas, an acute portal vein thrombosis, an acute bleeding requiring operative therapy and an acute renal insufficiency requiring hemodialysis and intensive care.

**Table 2:** Comparison of perioperative data between laparoscopic and open resection of patients with hepatic alveolar echinococcosis.

Variable	LH (n = 23)	OH (n = 70)	*p-value*
Type of resection, n (%)			
Hemihepatectomy right	2/23 (9)	17/70 (24)	*0*.*110*
Extended hemihepatectomy right	0/23 (0)	14/70 (20)	*0*.*021*
Hemihepatectomy left	0/23 (0)	8/70 (11)	*0*.*092*
Extended Hemihepatectomy left	0/23 (0)	2/70 (3)	*0*.*415*
Bisegmentectomy	4/23 (17)	9/70 (13)	*0*.*588*
Segmentectomy	1/23 (4)	4/70 (6)	*0*.*802*
Atypical resection	16/23 (70)	14/70 (20)	*<0*.*001*
Other	0/23 (0)	2/70 (3)	*0*.*415*
Operation time, mean minutes (SD)	127 (±60)	242 (±98)	*<0*.*001*
Blood loss, mean ml (SD)	307 (±327)	694 (±516)	*0*.*003*
Complications (Clavien-Dindo), n (%)	2/23 (9)	30/70 (43)	
1	0/23 (0)	1/70 (1)	*0*.*567*
2	1/23 (4)	8/70 (11)	*0*.*322*
3a	1/23 (4)	15/70 (21)	*0*.*016*
3b	0/23 (0)	4/70 (6)	*0*.*802*
4a	0/23 (0)	4/70 (6)	*0*.*244*
4b	0/23 (0)	0/70 (0)	*-*
5	0/23 (0)	0/70 (0)	*-*
Major complication (Clavien-Dindo ≥3b), n (%)	0/23 (0)	8/70 (11)	*0*.*322*
Length of hospital stay, mean days (SD)	4 (±2)	13 (±10)	*<0*.*001*
Postoperative treatment with albendazole, n (%)	17/23 (74)	64/70 (93)	*0*.*044*
Resection status, n (%)			
R0	16/23 (70)	39/70 (56)	*0*.*244*
R1	5/23 (22)	19/70 (27)	*0*.*609*
R2	0/23 (0)	3/70 (4)	*0*.*315*
Unknown	2/23 (9)	9/70 (13)	*0*.*594*
Recurrence, n (%)	0/23 (0)	3/70 (4)	*0*.*210*

LH, Laparoscopic hepatectomy; OH, open hepatectomy; SD, standard deviation

### Serology

**Fig 3:**
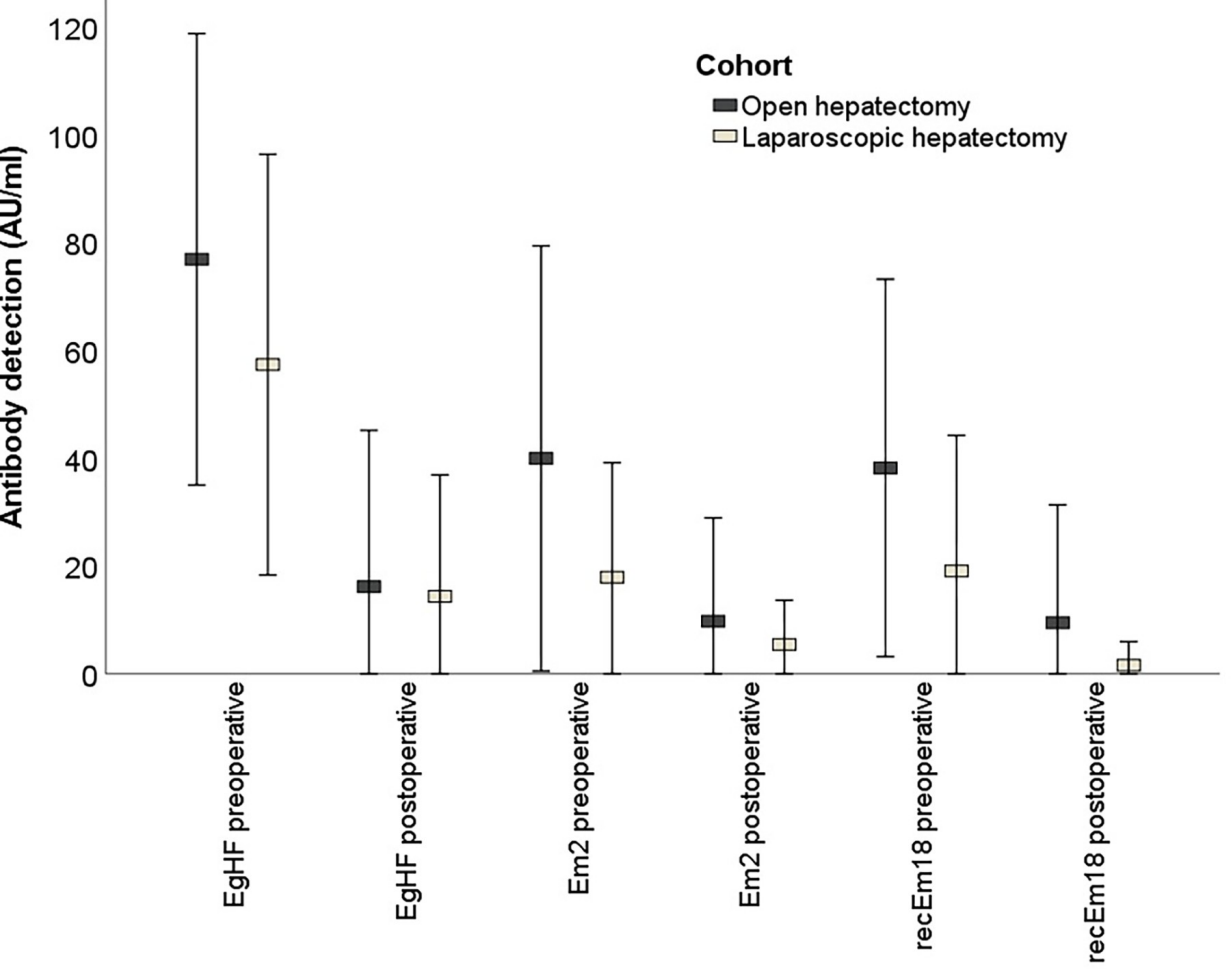
Boxplots for the pre- and postoperative serology of EgHF, Em2 and recEm18 (AU/ml). The bars show mean values and T-bars standard deviations respectively. A significantly lower postoperative antibody level is seen in all sampled antibodies. AU/ml, arbitrary units per milliliter; EgHF, Echinococcus granulosus hydatid fluid; Em2, Echinococcus multilocularis 2; recEm18, recombinant Echinococcus multilocularis 18.

Serum antibody levels (EgHF [n = 72/93, 77%], Em2 [n = 78/93, 84%] and recEm18 [n = 76/93, 82%]) were significantly lower within 12 months after curative resection. Due to missing data, in particular in the earlier cases where serology was not routinely used, not all patients could be examined. recEm18, which has the most clinical use of the mentioned antibodies, presented a negativization rate of 65.7% (n = 50/76) during postoperative course. This was apparent in LH cohort as well as in OH cohort and is displayed in Fig 3. LH cohort had lower mean preoperative values in antibodies than OH cohort (EgHF: 57 AU/ml vs. 77 AU/ml, *p = 0*.*064*; Em2: 18 AU/ml vs. 40 AU/ml, *p = 0*.*017*; recEm18: 19 AU/ml vs. 38 AU/ml, *p = 0*.*018*).

### Perioperative treatment with albendazole

Albendazole (ABZ) was administered as medical therapy in patients receiving pre- or postoperative benzimidazole therapy. In both cohorts a similar percentage of patients received preoperative medication with ABZ (LH 70% [n = 16/23] vs. OH 73% [n = 51/70], *p = 0*.*392*) (Table 1). Postoperatively, patients with LH received less often postoperative therapy (n = 17/23, 74%) compared with OH patients (n = 64/70, 93%, *p = 0*.*044*), as it is shown in Table 2. The mean duration of application of preoperative ABZ-medication was shorter in the LH cohort (3 months [SD ±2]) compared with patients from the OH cohort (5 months [SD ±9], *p = 0*.*529)*. Postoperative medical therapy in mean was not significantly shorter in patients treated laparoscopically (LH 15 months [SD ±10] vs. OH 22 months [SD ±25], *p = 0*.*684)*. In total, 10 patients (n = 10/93, 11%) developed adverse reactions to the medical therapy with ABZ. Increased liver transaminases were seen in 4 patients and 6 patients suffered from alopecia.

### Histopathological data

In the histopathological samples analyzed (n = 82/93, 88%), AE was confirmed in all cases, although PAS activity was only reported in 33/82 patients (40%). No significant differences were detected for the rate of R1-resections (LH 22% [n = 5/23] vs. OH 27% [n = 19/70], *p = 0*.*265*). The mean resection margin in R0 resected patients was also not significantly different (LH 0.3 cm [±0.4 cm, 13/16] vs. OH 0.7 cm [±1.1 cm, 34/39], *p = 0*.*605*) However, resection margin ranged from 0.0 cm to 5.0 cm in the OH cohort and from 0.0 cm to 2.0 cm in the LH cohort, and exact numbers were missing for 3 and 5 samples, respectively.

### Follow-up and recurrence

**Fig 4:**
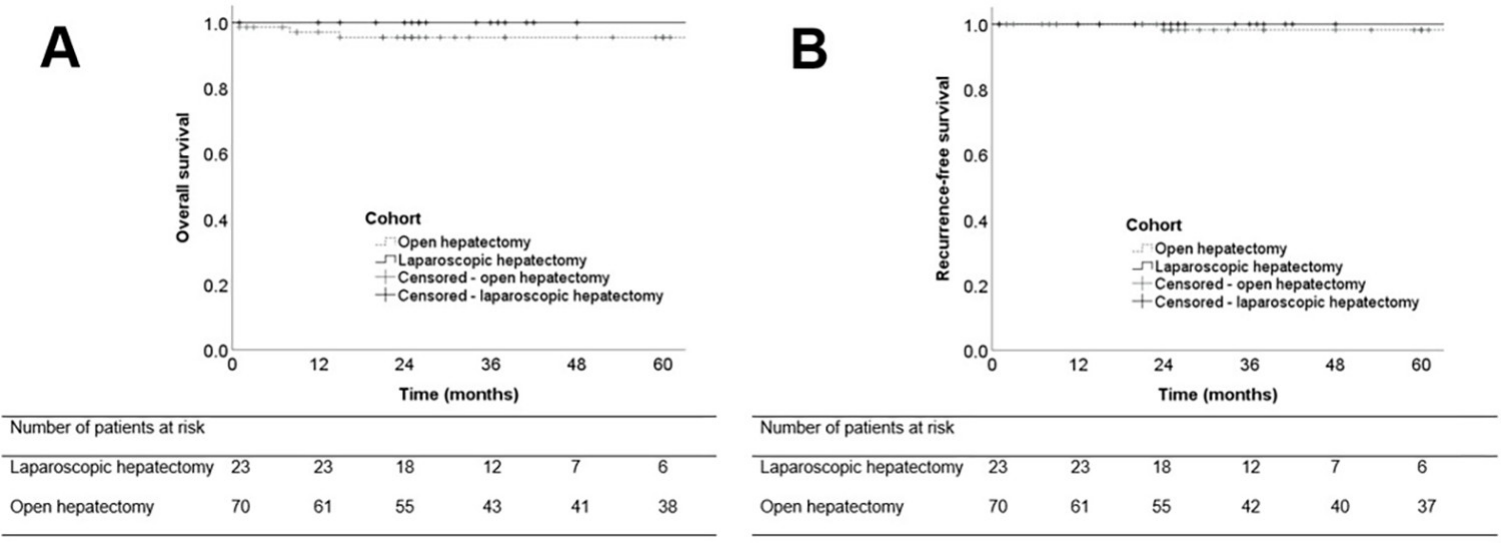
Overall survival (A) and recurrence-free survival (B) of patients undergoing surgical resection due to alveolar echinococcosis.

The 5-year overall survival showed no differences (LH 100% vs. OH 97%, *p = 0*.*264*), as it is seen in Fig 4A. Patients with LH had a distinct, but not significant lower recurrence rate (0% [n = 0/23] vs. 6% [n = 3/70], *p = 0*.*210*) during a mean follow-up of 55 months compared with a follow-up of 76 months in the OH cohort (*p = 0*.*245*). Fig 4B shows that the 5-year recurrence-free survival was not different (LH 100% vs. OH 96%, *p = 0*.*391*). However, recurrence was seen after a mean time of 68 months after resection. All patients with recurrence (n = 3/70, 4%) in the OH cohort recurred after minor resections. Whereas one patient had R0 resection, the other two patients had missing data because they were treated in the earlier days. Postoperative treatment with albendazole was administered at least in 2 patients, whereas the other patient had missing data. Additionally, one of the patients developed increasing liver enzymes and the treatment needed to be stopped after 5 months. In all patients recurrent lesions were apparent at the former resection margin. Mean diameter of lesions in patients with recurrence was 5.0 cm, compared to 7.0 cm in patients without recurrence (*p = 0*.*867*) and distant extrahepatic lesion was present in one patient. However, detailed pathological data of recurrence in OH cohort was incomplete (n = 2) due to missing reports.

### Subgroup analysis comparing PNM stage 1 patients

**Fig 5:**
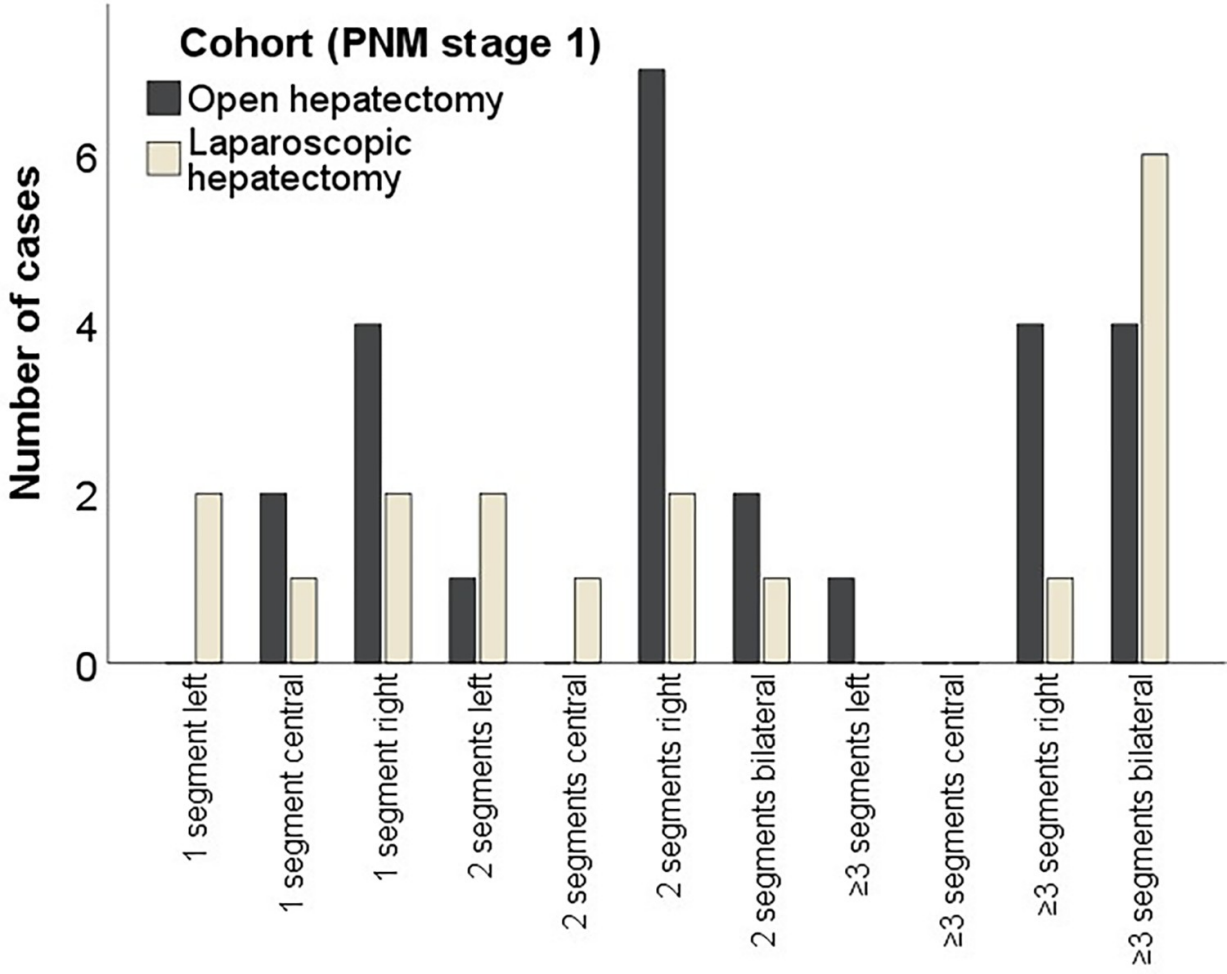
Location of hepatic AE lesions according to surgical approach of PNM stage 1 patients.

After subgroup analysis of all patients with PNM stage 1 showed that 18 patients (n = 18/43, 42%) were treated with laparoscopic approach, whereas 25 patients (n = 25/43, 58%) with limited disease stage had open hepatectomy (Table 3). The patients of the PNM stage 1 LH cohort had slightly more female patients (56% [n = 10/18] vs. 48% [n = 12/25], *p = 0*.*629*) and were older (61 years vs. 53 years, *p = 0*.*076*). Comparing mean maximal diameter of the AE lesion, the PNM stage 1 LH cohort had smaller lesions (3.3 cm vs. 6.0 cm, *p = 0*.*008*). Distribution pattern showed following segments affected by liver lesions in the LH cohort: One left-sided segment (n = 2/18, 11%), one central segment (n = 1/18, 6%), one right sided segment (n = 2/18, 11%), two left-sided segments (n = 2/18, 11%), two central segments (n = 1/18, 6%), two right-sided segments (n = 2/18, 11%), two bilateral segments (n = 1/18, 6%), more than three right-sided segments (n = 1/18, 6%) and more than three bilateral segments (n = 6/18, 33%). In contrast, in the OH cohort hepatic lesions were located in one central segment (n = 2/25, 8%), one right sided segment (n = 4/25, 16%), two left-sided segments (n = 1/25, 4%), two right-sided segments (n = 7/25, 28%), two bilateral segments (n = 2/25, 8%), more than three left-sided segments (n = 1/25, 4%), more than three right-sided segments (n = 4/25, 16%) and more than three bilateral segments (n = 4/25, 16%). Fig 5 shows that patients in the LH cohort were slightly more often treated for AE in left-lateral segments (*p = 0*.*081*), whereas patients in the OH cohort had more right liver resections (*p = 0*.*163*). The difference in operation time stayed significantly shorter with 120 minutes vs. 223 minutes (*p < 0*.*001*) in the subgroup analysis of PNM stage 1 patients. Postoperative major complication rate was low in both PNM stage 1 cohorts (PNM stage 1 LH 0% [n = 0/18] vs. PNM stage 1 OH 8% [n = 2/25], *p = 0*.*759*). Mean length of stay was shorter in PNM stage 1 LH cohort (4 days vs. 12 days, *p < 0*.*001*). R0-resection was performed in 67% (n = 12/18) in the PNM stage 1 LH cohort and 76% (n = 19/25) in the PNM stage 1 OH cohort respectively (*p = 0*.*569*). Mean resection margin of R0 resected patients was insignificant smaller in the PNM stage 1 LH cohort (PNM stage 1 LH 0.3 cm [±0.4 cm, 10/12] vs. PNM stage 1 OH 0.5 cm [±0.9 cm, 18/19], *p = 0*.*621*). Recurrence rate during follow-up was low (PNM stage 1 LH 0% [n = 0/18] vs. PNM stage 1 OH 8% [n = 2/25], *p = 0*.*682*).

**Table 3:** Comparison of demographic and perioperative clinical data between laparoscopic and open resection of PNM I staged patients with hepatic alveolar echinococcosis.

Variable	LH (n = 18)	OH (n = 25)	*p-value*
Gender, n (%)			*0*.*629*
Female	10/18 (56)	12/25 (48)	
Male	8/18 (44)	13/25 (52)	
Age, mean years (SD)	61 (±16)	53 (±16)	*0*.*076*
Maximal diameter of lesion, mean cm (SD)	3.3 (±1.9)	6.0 (±3.4)	*0*.*008*
Extrahepatic lesions, n (%)	0/18 (0)	0/25 (0)	*-*
Time from diagnosis to operation, mean months (SD)	4.4 (±5.5)	2.8 (±3.4)	*0*.*324*
Major operation, n (%)	0/18	13/25 (52)	*0*.*003*
Operation time, mean minutes (SD)	120 (±52)	223 (±52)	*<0*.*001*
Complications (Clavien-Dindo), n (%)	1/18 (6)	8/25 (28)	
1	0/18 (0)	0/25 (0)	*-*
2	0/18 (0)	3/25 (12)	*0*.*132*
3a	1/18 (6)	3/25 (12)	*0*.*132*
3b	0/18 (0)	2/25 (8)	*0*.*759*
4a	0/18 (0)	0/25 (0)	*-*
4b	0/18 (0)	0/25 (0)	*-*
5	0/18 (0)	0/25 (0)	*-*
Major complication (Clavien-Dindo ≥3b), n (%)	0/18 (0)	2/25 (8)	*0*.*759*
Length of hospital stay, mean days (SD)	4 (±2)	12 (±7)	*<0*.*001*
Preoperative treatment with albendazol, n (%)	11/18 (61)	17/25 (68)	*0*.*388*
Postoperative treatment with albendazol, n (%)	12/18 (67)	22/25 (88)	*0*.*163*
Resection status, n (%)			
R0	12/18 (67)	19/25 (76)	*0*.*506*
R1	4/18 (22)	3/25 (12)	*0*.*376*
R2	0/18 (0)	0/25 (0)	*-*
Unknown	2/18 (11)	3/25 (12)	*0*.*929*
Recurrence, n (%)	0/18 (0)	2/25 (8)	*0*.*682*

LH, Laparoscopic hepatectomy; OH, open hepatectomy; SD, standard deviation

## Discussion

In this single-center retrospective study of alveolar echinococcosis (AE) patients, laparoscopic hepatic resection of AE was found to be feasible and safe, with no signs of disease recurrence after mean follow-up of 55 months.

While both cohorts of laparoscopic and open resections had similar baseline characteristics in terms of gender, age, time to resection and preoperative benzimidazole (BZM) therapy with (ABZ), patients operated by laparoscopic resection had significantly lower P-stages, smaller hepatic tumors and no extrahepatic disease. This points to a potential selection bias towards less advanced and easier to resect patients in our study. In addition to the learning curve that comes with the implementation of a new surgical technique, it needs to be taken into account that advanced AE often grows along the central bile ducts requiring complex biliary reconstructions more difficult to perform with the laparoscopic approach [27]. Nevertheless, our subgroup analysis of PNM stage 1, where baseline characteristics of the laparoscopic resected patients approach those of the open resected patients even more, supports our results.

Similar to the steady increase of laparoscopic liver resections for other indications [16,28,29], we observed a continuous rise of laparoscopic AE resections over time with a shorter hospital stay and significantly lower complication rates reflecting the well-known benefits of laparoscopic liver surgery. Only one larger series of 13 laparoscopically resected AE-patients was published reporting a major complication rate of 7.7% [13]. Thus, our data might be also compared with the outcome of other cohorts of oncologic laparoscopic liver surgery, with major complication rates of around 11% [30]. Perioperative data of our cohort showed a significantly shorter operation time which is comparable to studies of laparoscopic resection of colorectal liver metastasis [31,32], also taking into account a potential bias towards easier resections.

Short operating times and low complication rates clearly result in significantly shorter hospital stays, which has already been demonstrated for oncologic laparoscopic liver resections [28,30,33]. Complication rates and length of stay of our open hepatectomy cohort were comparable with those from other centers performing AE-resections [9,10,34].

In our laparoscopic hepatectomy cohort fewer patients received postoperative ABZ therapy and if administered the duration of therapy was shorter than the 24 months postoperative treatment recommended by international standards [5,35]. Despite these recommendations, we did not observe the development of metastasis or recurrences after complete laparoscopic resection, but the rather short follow-up should be taken into consideration when interpreting these results. In contrast, the open hepatectomy cohort shows 3 patients with recurrence. However, these patients had either extent disease or where treated in the early days, where experience in AE treatment was lower. Furthermore, the resection margin status of two patients was not available, which makes it impossible to draw any conclusion for these patients. With the simultaneous improvement of disease activity evaluation by our serology [26] and our collected monocentric experience with very low recurrence rates after AE resection, we have now developed a disease surveillance approach based on radiological examinations and activity measurement by recEm18 serology guiding the indication and duration of postoperative ABZ treatment. This approach, which is based on retrospective data, has currently not been validated yet and clearly requires additional prospective validation.

Perioperative serum antibody levels against recEm18, Em2 and EgHF antigens were routinely analyzed during diagnostic work-up and follow-up in our center during the last years [22,26]. It allows a differentiation between Echinococcus species during diagnostic work-up [36] and due to the negativization after curative resection it may act as an alternative in disease surveillance. However, the possible role of recEm18 in disease surveillance needs further prospective studies. Therefore, assessment of recEm18 serum antibody levels is of most clinical use of all mentioned tests so far [8,22]. In line with the study by Gottstein et al. [26], all 3 ELISAs showed decreased values after resection suggesting a correlation with the load of parasites. Em2 has more cross-reactions with cystic echinococcosis [37] and is used as a diagnostic tool just in combination with EgHF and recEm18. The fact, that the LH cohort had lower values of recEm18 compared to the OH cohort might be explained by the smaller and less active lesions.

The rates of histologically margin-positive resections (R1) were not different between laparoscopic and open resection in our cohort. While conventional standards recommend to perform resections with a safety margin of 2 cm [5,11], others have reported that even lower margins of up to 1mm showed good long term outcomes in combination with anthelminthic therapy [38]. In line of the study by Hillenbrand et al., even patients with microscopically positive resection margins in our LH cohort did not develop disease recurrence during follow-up. While the slightly higher recurrence rate of 4% in our OH cohort is clearly in the range of published recurrence rates between 2% and 16% at 5–20 years after curative resection [9,11,14,19], the resection margin seemed to have no influence on disease recurrence. 5-year recurrence-free survival and overall survival rates of our patients were similar to the current literature after curative resection [9–11,14] and no differences between open or laparoscopic surgery could be detected.

Even though our study has clear limitations due to its retrospective design, missing histopathological data in patients with recurrence, which avoids a differentiation between recurrence and persistence of disease, a certain selection bias towards less advanced patients for the laparoscopic approach and a limited follow-up, we are the first western group presenting results of the largest cohort of laparoscopic AE resections so far. Laparoscopic early-stage AE resection is feasible and save with long-term recurrence-free survival.

## Conclusion

Laparoscopic resection of AE seems to be feasible and safe with no negative impact on perioperative outcomes, early disease recurrence or survival within a follow-up of mean 55 months. Therefore each patient should be evaluated by an experienced minimal invasive liver surgery team at an AE center with interdisciplinary treatment, eventually also allowing major laparoscopic AE resections in the future.
